# Knowledge and Awareness of Dental Practitioners About the Utilization of Endocrown in Post-endodontic Management

**DOI:** 10.7759/cureus.49838

**Published:** 2023-12-02

**Authors:** Ahmed A Madfa, Moazzy I Almansour, Asma F Alshammari, Nada M. Alenezi, Essa F. Alrashidi, Adel A. Aldhaban, Thoraya Aljohani, Faris A. Alshammari

**Affiliations:** 1 Department of Restorative Dental Science, College of Dentistry, University of Ha'il, Ha'il, SAU; 2 Department of Restorative Dental Science, College of Dentistry, University of Ha’il, Ha'il, SAU; 3 Dentistry, King Saud Medical City, Riyadh, SAU; 4 Dentistry, Rubue Aljazeera Medical Center, Ha'il, SAU; 5 Dentistry, Salmat Medical Group, Ha'il, SAU

**Keywords:** survey, endodontically treated teeth, dental practitioners, restorations, endocrown

## Abstract

Background: The objective of this study was to evaluate the knowledge, and awareness of dentists in Ha'il, Kingdom of Saudi Arabia, regarding the use of endocrown as post-endodontic restorations, utilizing an online questionnaire.

Methods: A cross-sectional study was carried out among dental practitioners working in Ha'il, Kingdom of Saudi Arabia. Dentists who practice in the Ha'il were included in the current study. The survey study involved a sample size of 245 participants. The researchers employed the snowball sampling technique in this investigation. The validated, closed-ended questionnaires were disseminated to the entire sample of selected dental practitioners using electronic mail. The initial section of the survey encompassed inquiries pertaining to the demographic characteristics of the participants, encompassing variables such as gender, years of professional experience, and workplace. The subsequent section of the survey focused on assessing the participants' knowledge and opinions regarding the endocrown technique. The Chi-square test was employed to assess the associations between categorical variables.

Results: The most of participants 228 (93.1%) had knowledge about endocrown and 94 (38.4%) of them received information from their educational institutions. Among the responses of the participants, 232 (94.7%) expressed their preference for utilizing endocrown restorations specifically for molar teeth. Moreover, 183 (74.7%) of respondents indicated that the endocrown is preferred when there is a restricted amount of inter-arch space available. A majority of respondents 152 (62.0%) indicated that the ferrule does not exert any influence on the endocrown. A majority of participants 135 (55.1%) expressed a preference for utilizing lithium disilicate ceramic in the fabrication of endocrown. The most of participants 209 (85.3%) opted to use resin cement for the purpose of cementation. The characteristics of gender, experience, and working place were found to have a significant relationship with the knowledge of the participants about endocrown (p <0.05).

Conclusions: The study participants need to enhance their knowledge and awareness pertaining to the utilization of endocrowns as a post-endodontic treatment.

## Introduction

The management of post-endodontic treatment involves the restoration of the tooth, with the aim of protecting and preserving the residual tooth structure, while also ensuring appropriate aesthetics, form, and function [1]. The primary objective of this management approach is to achieve the least invasiveness in teeth preparation while ensuring the maximum preservation of the surrounding tissues of the restored teeth [2]. The objective is to attain a mechanically stabilized tooth-restoration complex with a suitable surface for adhesion. Nevertheless, the restoration of teeth that have undergone endodontic treatment and have experienced significant damage to the crown poses considerable difficulty in the field of clinical practice. Endodontically treated teeth experience a reduction in their mechanical characteristics, resulting in increased fragility and susceptibility to fractures mostly due to the loss of structural integrity of the tooth [3]. The removal of pulp tissue and subsequent loss of vitality have a detrimental effect on the neurosensory feedback system. The potential consequence of this is a decrease in the physical qualities of dentin, such as the modulus of elasticity and fracture resistance. These changes may ultimately contribute to a higher rate of failure in teeth that have undergone endodontic treatment [4]. The access cavity, enlargement of the pulp chamber, and loss of dentin-supporting walls that occur during endodontic treatment have a substantial impact on the fracture resistance of teeth. These factors contribute to an increase in cuspal deflection and the likelihood of cuspal fracture. Hence, the preservation and maintenance of tooth structure are of utmost importance in the treatment of endodontically treated teeth [5].

The prompt completion of an effective sealing procedure on the root canal system following root canal treatment plays a crucial role in determining the overall success rate of the treated tooth over an extended period of time. The occurrence of teeth exhibiting insufficient coronal restorations is notably greater (54.3%) compared to teeth having satisfactory coronal restorations (34.7%). The user has provided a numerical reference. There exist several methods for the restoration of teeth subsequent to root canal treatment, including direct filling with composite resin, inlay restoration, onlay restoration, full crown restoration, and post-core crown restoration [6]. The selection of either direct or indirect restoration is contingent upon the extent of the defect's severity. In conventional dental practice, the preferred approach for addressing extensive defects in tooth structure involves the use of indirect restorations, specifically crowns, to replace the affected tooth.

When seeking dental restoration, patients express a nee

d for three key factors: longevity and durability, aesthetic appeal, and minimal invasive tooth preparation. In the past, traditional post and core crowns were widely regarded by dentists as the sole option for the restoration of extensively damaged teeth that had undergone endodontic treatment. Nevertheless, due to the progress in adhesive dentistry, the implementation of endocrown, a conservative restoration consisting of a single unit, has emerged as a feasible alternative treatment approach to the traditional method involving post/core and crown. The adhesive endodontic crown known as the endocrown was initially introduced by Bindl and Mormann in 1999 [7]. The authors provided a definition for it, referring to a complete porcelain crown that is permanently affixed to posterior teeth that have undergone endodontic treatment. The optimal approach for achieving long-term clinical success in the restoration of severely damaged teeth that have undergone endodontic therapy is characterized by minimal invasive preparations, maximal preservation of tissue, avoidance of excessive treatment, and the capability to perform additional root canal treatment if issues arise [3]. The previously established benchmarks for the restoration of severely damaged teeth that have undergone endodontic therapy provide a rationale for incorporating the use of endocrown in the prosthetic treatment strategy for such teeth [3]. Furthermore, the utilization of endocrown as an alternative therapy modality for endodontically treated teeth is supported by several clinical scenarios, such as calcified root canals, shattered instruments, and limited canals [8].

The endocrown technique involves the use of the pulp chamber and the cavity margin, without the need for root canal intervention. As a consequence, there is a reduction in the amount of tooth structure that needs to be removed, while also facilitating the establishment of macro-micro mechanical retention between the pulp chamber walls and the adhesive cementation system [9]. The preparation of an endocrown involves the simultaneous preparation of parallel occlusal surfaces. This feature offers enhanced resilience to stress and a supragingival cervical margin. The maintenance of periodontium health and the facilitation of impression-taking are supported by this practice [9]. The occlusal aspect of the endocrown exhibits a notable degree of protection against fractures. The thickness of the crown exhibits variability ranging from 3 to 7 mm greater than that of the traditional crown, as indicated by reference [3]. Hence, there is no requirement for any supplementary macro-retentive preparation or ferrule. The use of a ferrule in the preparation of an endocrown does not yield a substantial improvement in fracture resistance compared to an endocrown prepared without a ferrule [9]. The fabrication of endocrown restorations involves the utilization of either a traditional heat-pressed method or a computer-aided design/computer-aided manufacture (CAD/CAM) system. The utilization of CAD/CAM technology is prevalent in contemporary dental environments. One notable benefit is its capacity to deliver superior restoration outcomes in a significantly condensed time frame during chair-side procedures. Since the advent of CAD/CAM, numerous associated materials have been employed [9,10]. The materials frequently employed for the fabrication of endocrown include leucite-reinforced ceramics, lithium disilicate-reinforced ceramics, and monolithic zirconia ceramics. Lithium disilicate is widely employed as a material of choice for the fabrication of endocrown. The object in question possesses commendable aesthetic qualities and demonstrates satisfactory mechanical durability, as indicated by sources [11,12]. In recent times, there has been a notable advancement in the development and utilization of resin composite materials. Specifically, nanoceramic resin restorative materials have emerged as a significant innovation. These materials possess a modulus of elasticity that closely resembles that of dentine, exhibit reduced crack propagation, and have superior fracture resistance in comparison to ceramics [3]. Nevertheless, the occurrence of microleakage is a significant challenge in resin composite materials [10]. In addition, it exhibits lower strength and fracture resistance compared to disilicate lithium glass-ceramic [11]. The utilization of endocrown in contemporary clinical settings has demonstrated numerous advantages in comparison to traditional posts and cores. The primary objective of endocrown is to retain the biomechanical integrity of teeth that have undergone endodontic treatment, while also preserving the greatest possible amount of tooth structure for the purpose of bonding [3]. The utilization of this method leads to minimum loss of tooth structure, enhanced mechanical characteristics, a reduced number of clinical procedures, and decreased expenses [12]. The aesthetic characteristics are also exceptional. Moreover, they offer a notable benefit in cases when posts are not recommended due to the presence of short or narrow canals. In light of the growing prevalence of endocrown restorations within the dental field, a pertinent inquiry arises as to whether physicians ought to contemplate the utilization of endocrown as an alternative to traditional treatments including intra-radicular posts. Numerous in vitro investigations have been conducted to assess the efficacy of endocrown in comparison to conventional post-retained crowns. According to many studies, it has been stated that endocrown have a fracture strength that is either equivalent to or even surpasses that of traditional crowns, particularly when utilized in molars, premolars, and even incisors [3,12]. Nevertheless, based on the available information, there remains a limited amount of clinical data documented in the literature. However, there is a sufficient amount of in vitro research accessible that reports on the fracture strength of endocrown. As a result, observational studies and the opinions of clinicians take this matter into consideration [13].

In order to effectively address the patient's requirements and achieve sustainable outcomes in restorative dentistry, it is imperative for dentists to possess comprehensive knowledge of all potential dental treatment alternatives. The available solutions are determined by the structural integrity of the tooth, the aesthetic preferences of the patients, and the need to protect the remaining tooth structure. Nevertheless, there exists a scarcity of accessible data pertaining to the utilization of endocrowns in dental restorations. So far, there has been an absence of research conducted to assess the level of knowledge and perspectives among dentists in Ha'il regarding the utilization of endocrown in dental procedures. The objective of this study is to evaluate the knowledge and awareness of dentists working in Ha'il, Kingdom of Saudi Arabia, about the use of endocrown for the restoration of teeth that have had endodontic treatment.

## Materials and methods

This cross-sectional study was conducted among dentists practicing in Ha'il, Kingdom of Saudi Arabia. This survey was undertaken between June 2022 and December 2022, following the acquisition of ethical approval from the research council of the University of Ha'il. The study protocol (NO: H2022-326), was approved by the institutional review board of our university.

Upon conducting a thorough review of the existing literature, the questionnaires included in this study were meticulously devised. The questionnaire form's reliability was validated during a pilot testing phase that included the participation of 30 practitioners. The pertinence of the questionnaire to the subject matter of the survey was verified by faculty members from the Department of Restorative Dental Science at the College of Dentistry, University of Ha'il, Kingdom of Saudi Arabia, in conjunction with specialists in the field. 

The estimated sample size for the study was found to be 250 using the sample size calculator provided by OpenEpi®. The statistical power was set at 84% and the significance level (α) was set at 5%. This study involves the participation of dental experts. The study comprised a sample of 300 dentists from Ha'il province, selected through a snowball sampling method. The current study included dentists practicing in the region of Ha'il. These participants were then given a questionnaire that was designed to be self-explanatory. A total of 275 questionnaires were collected, of which 245 were considered eligible for inclusion in the study.

Practitioners were subjected to the administration of questionnaires to collect data derived from their knowledge and awareness about endocrown. Prior to the commencement of data collection, participants were required to express their informed consent. The researchers employed the snowball sampling technique in this investigation. A web-based self-administered questionnaire, consisting of two parts, was disseminated in English. The questionnaire encompassed a concise elucidation of the study's objective, the approach employed for data gathering, and a series of closed-ended inquiries. The first part of the questionnaire consisted of inquiries pertaining to the socio-demographic attributes of the participants such as gender, experience, and workplace. The next part of the questionnaire comprised a series of eight inquiries following a specific structure. The following section of the survey collected data regarding the knowledge and awareness related to endocrown. To ensure the preservation of confidentiality, the responses were rendered entirely anonymous.

The questionnaire was transformed into an electronic format by utilizing Google Forms, a web-based survey generator that is available free of charge. The questionnaire was prepared and subsequently disseminated through email and several social media sites. The hyperlink to the questionnaire remained accessible for a duration of six months. Two subsequent reminders to participate were given at intervals of three weeks each following the initial invitation.

The statistical analysis was conducted using Statistical Package for the Social Sciences (SPSS) Software (Version 25.0). The research variables were represented for frequency distribution and cross-tabulation. The chi-square test was employed to assess the potential association between knowledge and awareness about endocrown and variables such as gender, experience, and working place. The level of significance was established at 5% (p < 0.05).

## Results

The questionnaire encompassed a sample size of 245 individuals. Of the participants, 137 (55.9%) were identified as males while 108 (44.1%) were identified as females. Additionally, 142 (58.0%) of the participants had completed their education and had less than 5 years of professional experience. Furthermore, 68 (27.8%) of the participants had completed their education and possessed 5-10 years of professional experience. The data reveals that 109 (44.5%) of the participants were employed in the government sector, while 81 (33.1%) were employed in the private sector. Additionally, 55 (22.4%) of the participants were residents of the university.

Table 1 displays the distribution of participants according to their knowledge and awareness of the endocrown. When inquiring with the participants regarding their familiarity with the concept of endocrown, it was found that 228 (93.1%) of them had knowledge about endocrown. A total of 94 (38.4%) of respondents reported receiving information about endocrown from their college, while 75 (30.6%) indicated that they acquired knowledge about endocrown through online sources. The results of the study reveal that a significant majority of the participants, specifically 232 (94.7%), expressed the view that endocrown restorations are appropriate for molar teeth. The utilization of endocrown as a treatment option for cases involving moderate loss of tooth structure was reported by 111 (45.3%) of the study participants. However, it was observed that 85 (34.7%) of the subjects exhibited indications of significant loss of tooth structure. The utilization of endocrown as a clinical treatment option is favored above the usual approach of post and core restoration in cases where there is a limited amount of inter-arch space, as indicated by 183 (74.7%) of respondents. Approximately 152 (62.0%) of respondents expressed the belief that the presence of a ferrule does not have an impact on the performance of an endocrown. According to the survey results, a majority of the participants 135 (55.1%) said that the preferred material for endocrown fabrication is lithium disilicate ceramic. This was followed by zirconia, which was chosen by 78 (31.8%) of the respondents. The findings of the study indicate that a significant proportion of the participants 209 (85.3%) expressed their preference for utilizing adhesive resin cement as the optimal choice for cementation in endocrown procedures.

**Table 1:** Distribution of subjects based on knowledge and awareness about endocrown

Variables		Frequency	Percent
Are you knowledgeable about the concept of endocrown?	Yes	228	93.1%
	No	17	6.9%
If yes, from where you got the information?	College	94	38.4%
	Internet	75	30.6%
	Textbook	53	21.6%
	Conference and workshop	23	9.4%
The endocrown restoration is used for	Anterior teeth	2	0.8%
	Premolars	11	4.5%
	Molars	232	94.7%
What is the indication of using endocrown?	Extensive loss of tooth structure	85	34.7%
	Moderate loss of tooth structure	111	45.3%
	Minimum loss of tooth structure	49	20%
In which clinical scenario is the utilization of endocrown favored above the standard approach of post and core restoration?	Enough inter-arch space	62	25.3%
	Limited inter-arch space	183	74.7%
Do you need ferrule effect for endocrown?	Yes	93	38.0%
	No	152	62.0%
The preferred material for the fabrication of endocrown is	Nanocomposite resin	13	5.3%
	lithium disilicate	135	55.1%
	Zirconia	78	31.8%
	Fieldspathic porcelain	19	7.8%
What type of cement is used for endocrown?	Zinc phosphate	14	5.7%
	Glass ionomer	22	9.0%
	Adhesive resin cement	209	85.3%

A chi-square test was conducted to examine the relationship between knowledge and awareness, and several participant characteristics including gender, experience, and working place. The results indicated a statistically significant link (p < 0.05) between these parameters, as shown in Table 2.

**Table 2:** Association of knowledge with gender, experience, and workplace

Variables		Gender			Experience				Workplace			
		M	F	p-value	< 5 y	5-10 y	> 10	p-value	Educ.	Gov.	Private	p-value
Are you knowledgeable about the concept of endocrown?	Yes	130 (53.1)	98 (40.0)	.155	139 (56.7)	63 (25.7)	26 (10.5)	.000	77 (31.4)	102 (41.6)	49 (20.0)	.059
	No	7 (209)	10 (4.1)		3 (1.2)	5 (2.0)	9 (3.7)		4 (1.6)	7 (2.9)	6 (2.4)	
If yes, from where you got the information	College	57 (23.3)	37 (15.1)	.016	64 (26.1)	23 (9.4)	7 (2.8)	. .000	37 (15.1)	36 (14.7)	21 (8.6)	.016
	Internet	41 (16.7)	34 (13.9)		42 (17.1)	22(9.0)	11 (4.5)		26 (10.6)	29 (11.8)	20 (8.2)	
	Textbook	30 (12.2)	23 (9.4)		27 (11.0)	19 (7.8)	7 (2.8)		14 (5.7)	31 (12.7)	8 (3.3)	
	Conference and workshop	9 (3.7)	14 (5.7)		9 (3.7)	4 (1.6)	10 (4.1)		4 (1.6)	13 (5.3)	6 (2.4)	
The endocrown restoration is used for	Anterior teeth	0 (0)	2 (0.8)	.033	1 (0.4)	1 (0.4)	0 (0)	034	1 (0.4)	0 (0)	1 (0.4)	.124
	Premolars	4 (1.6)	7 (2.9)		4 (1.6)	3 (1.2)	4 (1.6)		5 (2.0)	3 (1.2)	3 (1.2)	
	Molars	133 (54.3)	99 (40.4)		137 (55.9)	64 (26.1)	31 (12.6)		75 (30.6)	106 (43.3)	51 (20.8)	
What is the indication of using endocrown?	Extensive loss of tooth structure	48 (19.6)	37 (15.1)	.011	47 (19.2)	23 (9.4)	15 (6.1)	025	29 (11.8)	39 (15.9)	17 (6.9)	.018
	Moderate loss of tooth structure	52 (21.2)	59 (24.1)		71 (29.0)	29 (11.8)	11(4.5)		37 (15.1)	52 (21.2)	22 (9.0)	
	Minimum loss of tooth structure	16 (6.5)	9 (3.7)		10 (4.1)	10 (4.1)	5 (2.0)		9 (3.7)	7 (2.9)	9 (3.7)	
In which clinical scenario is the utilization of endocrown favored above the standard approach of post and core restoration?	Enough inter-arch space	35 (14.3)	27 (11.0)	.117	35 (14.3)	13 (5.3)	14 (5.7)	.029	24 (9.8)	28 (11.4)	10 (4.1)	.027
	Limited inter-arch space	102 (41.6)	81 (33.1)		107 (43.7)	55 (22.4)	21 (8.5)		57 (23.3)	81 (33.1)	45 (18.4)	
Do you need ferrule effect for endocrown?	Yes	55 (22.4)	38 (15.5)	.077	48 (19.6)	26 (10.6)	19 (7.7)	.010	36 (14.7)	38 (15.5)	19 (7.8)	.032
	No	82 (33.5)	70 (28.6)		94 (38.4)	42 (17.1)	16 (6.5)		45 (18.4)	71 (29.0)	36 (14.7)	
The preferred material for the fabrication of endocrown is	Nanocomposite resin	8 (3.3)	5 (2.0)	.035	2 (0.8)	8 (3.3)	3 (1.2)	.004	3 (1.2)	5 (2.0)	5 (2.0)	.004
	lithium disilicate	81 (33.1)	54 (22.0)		75 (30.6)	41 (16.7)	19 (7.7)		39 (15.9)	62 (25.3)	34 (13.9)	
	Zirconia	37 (15.1)	41 (16.7)		52 (21.2)	13 (5.3)	13 (5.3)		29 (11.8)	35 (14.3)	14 (5.7)	
	Fieldspathic porcelain	11 (4.5)	8 (3.3)		13 (5.3)	6 (2.4)	0 (0)		10 (4.1)	7 (2.9)	2 (0.8)	
What type of cement is used for endocrown?	Zinc phosphate	8 (3.3)	6 (2.4)	.094	2 (0.8)	9 (3.7)	3 (1.2)	006	1 (0.4)	7 (2.9)	6 (2.4)	.018
	Glass ionomer	11 (4.5)	11 (4.5)		8 (3.3)	7 (2.9)	7 (2.8)		8 (3.3)	8 (3.3)	6 (2.4)	
	Adhesive resin cement	118 (48.2)	91 (37.1)		132 (53.9)	52 (21.2)	25 (10.1)		72 (29.4)	94 (38.4)	43 (17.6)	

## Discussion

The utilization of endocrown in clinical environments has demonstrated several benefits in contrast to the post-core technique, which has conventionally been considered the favored approach for restoring teeth that have received endodontic therapy [14]. The primary aim of employing the endocrown approach is to preserve the pre-existing tooth structure that can be utilized for bonding purposes, while simultaneously guaranteeing the biomechanical stability of teeth that have undergone endodontic treatment and may possess reduced structural integrity. The endocrown has been demonstrated to have enhanced mechanical qualities, leading to minimal tooth structure loss, reduced clinical interventions, decreased costs, and enhanced cosmetic results [15-20]. Therefore, the objective of this cross-sectional study was to assess the level of knowledge and opinions of dental practitioners on the utilization of endocrown as a s a post-endodontic treatment technique.

The findings of the current survey revealed that the primary means of acquiring information on endocrown was through college, which contradicts the findings of Al Moaleem et al. [21]. Their study reported that 45.97% of participants indicated that they obtained information about endocrown from friends, while only 25.04% leaned on college as a source of information. The conflict may arise from the inclusion of samples in the previous study, which consisted of volunteers from Saudi Arabia and other countries with varying degrees of expertise and experience.

The endocrown can be performed on all teeth, although it is advisable to limit its application to molars because of the differing masticatory forces experienced by premolars compared to molars [7]. In the current investigation, when questioned regarding the indications for using endocrowns in the field of endodontics, a significant proportion of participants conveyed the perspective that endocrowns are mostly suitable for application in molars. Nevertheless, there was limited belief regarding its application specifically for premolar and anterior teeth. The findings of this study align with a prior investigation conducted in Riyadh, Saudi Arabia, which emphasized the preference for utilizing endocrowns as a therapeutic technique for molars [22]. This study is also consistent with the findings of Rasidi and Priscilla, who observed that 83.33% of the participants expressed that endocrown restorations are appropriate for molar teeth [23]. Moreover, Al Moaleem et al. revealed that 61.23% of the participants expressed that endocrown restorations are appropriate for molar teeth [21]. According to a study conducted by Deepak and Nivedhitha, a majority of the participants, specifically 52.1%, expressed the belief that endocrown restorations are suitable for molar teeth [24]. According to Belleflamme et al., the utilization of endocrowns may be a viable option for the restoration of molars and premolars that have suffered extensive damage, even in cases where occlusal risk factors such as bruxism or unfavourable occlusal relationships are present [25].

In the present study, approximately 45.3% of participants expressed the belief that the utilization of endocrowns is appropriate in cases with moderate loss of tooth structure. In a study conducted by Deepak and Nivedhitha, it was shown that 34.5% of the participants expressed belief in the proper use of endocrown for cases with substantial loss of coronal tooth structure [24]. Additionally, 37.3% of the participants held this belief when more than half of the crown structure was destroyed. Rasidi and Priscilla found that when asked about the clinical application of endocrown for loss of tooth structure, 42.5% of dental practitioners responded with 'loss of three walls', 35.0% responded with 'loss of two walls', and only 22.5% responded with 'loss of one wall' [23].

The efficacy of endocrown restorations is impacted by the luting technique employed, and it is imperative to ensure a strong connection in order to optimize the mechanical properties and longevity of the restoration during masticatory activities [26]. The current study's findings indicate that a notable percentage of dental practitioners exhibit a preference for resin cement as their preferred material for the process of cementation. The findings of this study align with the recent research conducted by Shindhuja et al., which concluded that the use of resin-luting cement is preferred by most of the participants [27]. Additionally, the study conducted by Deepak and Nivedhitha, revealed that a significant proportion of dental practitioners indicated resin cement as their choice [24]. Sevimli et al. did a review that suggests that resin composite cements are the preferred materials for cementing endocrowns [3]. However, Rasidi and Priscilla revealed that a significant proportion of dental practitioners selected Type 1 GIC as their preferred cement material for endocrowns [23]. Nevertheless, a limited number of dental professionals provided responses indicating resin composite as the preferred material.

Lithium disilicate is widely employed as a primary material in the fabrication of endocrowns. The object possesses favorable aesthetic qualities and demonstrates sufficient mechanical durability, as indicated by sources [11,13]. The findings of the present study indicate that most of the participants (63.46%) displayed a preference for employing lithium disilicate-based ceramics as the preferred material for endocrown restorations. The present analysis is consistent with a prior study conducted in Saudi Arabia, which found that the favored material for endocrown was lithium disilicate [18,22]. The study conducted by Wahab et al. investigated the material preferences for endocrown among dentists in Jordan, and their findings aligned with the previously described research [16].

Regarding the ferrule effect, a notable majority of participants (62.0%) maintain the perspective that the existence of a ferrule does not exert any influence on the outcome of therapy. A previous study conducted in Saudi Arabia revealed that a significant majority of participants, over 80%, had the belief that the use of ferrule can enhance the ability of endodontically treated teeth to resist fractures [28]. A previous study examined the effect of including a ferrule in the use of an endocrown and found that it did not significantly influence clinical outcomes [9]. In a study conducted by Rasidi and Priscilla, it was shown that a majority of dental practitioners (88.33%) responded affirmatively when asked about the significance of ferrule, while just a minority (11.67%) responded negatively [23].

Endocrowns are commonly recommended for endodontically treated teeth that have a reduced crown height and an appropriate depth of the pulp chamber [8]. The findings of our research demonstrate that a significant proportion of dental practitioners (74.7%) exhibit a preference for utilizing endocrowns as the principal approach for the rehabilitation of teeth that have received endodontic treatment, particularly in cases where there exists a restricted inter-occlusal dimension. This finding is consistent with previous studies conducted in Riyadh, Saudi Arabia, that have emphasized the use of endocrowns as the favored treatment choice for cases with insufficient inter-occlusal space [28]. 

Based on the current study findings, imply that most dental professionals such as females, participants had completed their education and possessed 5-10 years of professional experience and participants were affiliated with educational institutions that adhered to evidence-based treatment approaches for endodontically treated teeth [27,28].

Several limitations should be taken into account while considering this study. The limited sample size may not sufficiently capture the features of the entire population. Furthermore, the failure to evaluate the dependency on files and the disregard for variances among operators were not well addressed. It is recommended to undertake a comprehensive multicenter study to assess the actual efficacy of endocrown therapy and its association with cosmetic results.

## Conclusions

Based on the limitations inherent in this study, it could be concluded that the participants, regardless of their gender, experience, and workplace, exhibited a low knowledge of the use of endocrown. It is apparent that the participants would benefit from more knowledge and awareness of the utilization of endocrown as a means of post-endodontic treatment. We recommend carrying out continuing dental education for dental practitioners to enhance their understanding of the appropriate utilization and indications of endocrown.

## References

[1] Robbins JW (2002). Restoration of the endodontically treated tooth. Dent Clin North Am.

[2] Bejoy BM, Anitha S, George L, Mathew J, Paul S, Joy A (2021). Conservative bonded restoration (an alternative to full coverage crown): a case report on endocrown. Conserv Dent Endod J.

[3] Sevimli G, Cengiz S, Oruc MS (2015). Endocrowns: review. J Istanb Univ Fac Dent.

[4] Sedrez-Porto JA, Sarkis-Onofre R, de Moraes AP, Correa MB, Cenci MS, Pereira-Cenc T (2017). Knowledge and attitudes of students and dentists about the use and cementation of intra-radicular posts. Braz Dent Sci.

[5] Song M, Park M, Lee CY, Kim E (2014). Periapical status related to the quality of coronal restorations and root fillings in a Korean population. J Endod.

[6] Lin CL, Chang YH, Pai CA (2011). Evaluation of failure risks in ceramic restorations for endodontically treated premolar with MOD preparation. Dent Mater.

[7] Bindl A, Mörmann WH (1999). Clinical evaluation of adhesively placed cerec endo-crowns after 2 years--preliminary results. J Adhes Dent.

[8] Dogui H, Abdelmalek F, Amor A, Douki N (2018). Endocrown: an alternative approach for restoring endodontically treated molars with large coronal destruction. Case Rep Dent.

[9] Tzimas K, Tsiafitsa M, Gerasimou P, Tsitrou E (2018). Endocrown restorations for extensively damaged posterior teeth: clinical performance of three cases. Restor Dent Endod.

[10] Ghajghouj O, Taşar-Faruk S (2019). Evaluation of fracture resistance and microleakage of endocrowns with different intracoronal depths and restorative materials luted with various resin cements. Materials (Basel).

[11] Guo J, Wang Z, Li X, Sun C, Gao E, Li H (2016). A comparison of the fracture resistances of endodontically treated mandibular premolars restored with endocrowns and glass fiber post-core retained conventional crowns. J Adv Prosthodont.

[12] Magne P, Carvalho AO, Bruzi G, Anderson RE, Maia HP, Giannini M (2014). Influence of no-ferrule and no-post buildup design on the fatigue resistance of endodontically treated molars restored with resin nanoceramic CAD/CAM crowns. Oper Dent.

[13] Carvalho AO, Bruzi G, Anderson RE, Maia HP, Giannini M, Magne P (2016). Influence of adhesive core buildup designs on the resistance of endodontically treated molars restored with lithium disilicate CAD/CAM crowns. Oper Dent.

[14] Sun J, Ruan W, He J, Lin X, Ci B, Yin S, Yan W (2019). Clinical efficacy of different marginal forms of endocrowns: study protocol for a randomized controlled trial. Trials.

[15] Abtahi S, Alikhasi M, Siadat H (2022). Biomechanical behavior of endocrown restorations with different cavity design and CAD-CAM materials under a static and vertical load: a finite element analysis. J Prosthet Dent.

[16] Wahab FD, Mahasneh SA, Sawair FA, Hamdan MA, Hattar SN, Al-Rabab’ah MA (2016). Restoration of root filled teeth; current opinions and techniques. Prim Dent J.

[17] Govare N, Contrepois M (2020). Endocrowns: a systematic review. J Prosthet Dent.

[18] El-Damanhoury HM, Haj-Ali RN, Platt JA (2015). Fracture resistance and microleakage of endocrowns utilizing three CAD-CAM blocks. Oper Dent.

[19] Aktas G, Yerlikaya H, Akca K (2018). Mechanical failure of endocrowns manufactured with different ceramic materials: an in vitro biomechanical study. J Prosthodont.

[20] Fages M, Bennasar B (2013). The endocrown: a different type of all-ceramic reconstruction for molars. J Can Dent Assoc.

[21] Al Moaleem MM, Al Ahmari NM, Alqahtani SM (2023). Unlocking endocrown restoration expertise among dentists: insights from a multi-center cross-sectional study. Med Sci Monit.

[22] Alotaiby F, Aldulaijan J, Alotaibi M (2021). Endocrowns: a retrospective study among Riyadh Elm University dental clinics. Int J Community Med Public Health.

[23] Rasidi MQ, Anthony SD, Prabhu Prabhu, D D (2021). Knowledge, attitude, and practice of endocrown in post-endodontic management among general practitioners. J Contemp Issues Business Government.

[24] Deepak S, Nivedhitha MS. (2017). Endocrown-post endodontic restoration-a questionnaire survey. J Pharm Sci Res.

[25] Belleflamme MM, Geerts SO, Louwette MM, Grenade CF, Vanheusden AJ, Mainjot AK (2017). No post-no core approach to restore severely damaged posterior teeth: an up to 10-year retrospective study of documented endocrown cases. J Dent.

[26] Gregor L, Bouillaguet S, Onisor I, Ardu S, Krejci I, Rocca GT (2014). Microhardness of light- and dual-polymerizable luting resins polymerized through 7.5-mm-thick endocrowns. J Prosthet Dent.

[27] Shindhuja N, Eazhil. R, A. Harish Raghav (2023). Assessment of awareness, knowledge, and attitude towards endocrowns among dental practitioners in Chennai - a questionnaire study. Int J Chem Biochem Sci.

[28] Soliman M, Alshamrani L, Yahya B, Alajlan G, Aldegheishem A, Eldwakhly E (2021). Monolithic endocrown vs. hybrid intraradicular post/core/crown restorations for endodontically treated teeth; cross-sectional study. Saudi J Biol Sci.

